# Supplementary material for: Single cell sequencing reveals endothelial plasticity with transient mesenchymal activation after myocardial infarction
Source: Nat Commun. 2021 Jan 29;12:681. doi: 10.1038/s41467-021-20905-1 (PMC7846794; doi:10.1038/s41467-021-20905-1)
Supplement: Supplementary file 1 — Supplementary Information [file 41467_2021_20905_MOESM1_ESM.pdf]

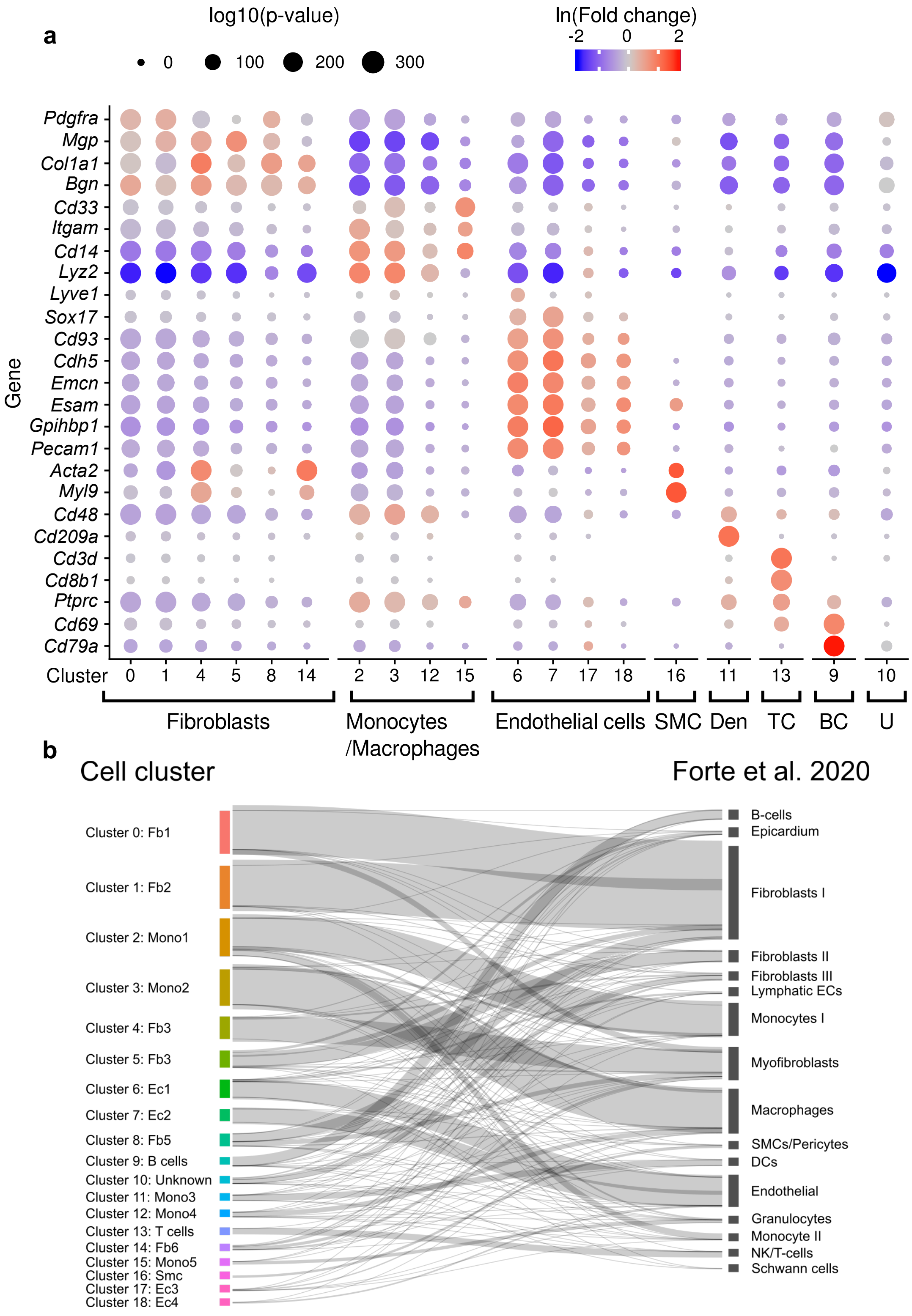

Supplementary Figure 1 – Single cell sequencing captures various cell types

a Dot plot representing marker for various cardiac cell types. Cell clusters were assigned to B-cells (9), endothelial cells (6,7,17,18), fibroblasts (0,1,4,5,8,14), monocytes (2,3,11,12,15), smooth muscle cells (SMC) (16) and T-cells (13). Cluster 10 (unknown; U) could not assign clearly based on presented markers. Fold-changes (blue to red) and p-values (dot size) were calculated with Seurat's 'FindAllMarkers' function. **b** Sankey plot showing correspondence between the clustering as shown in Figure 1a and the original clustering and cell type annotation performed in *Forte et al. 2020.*

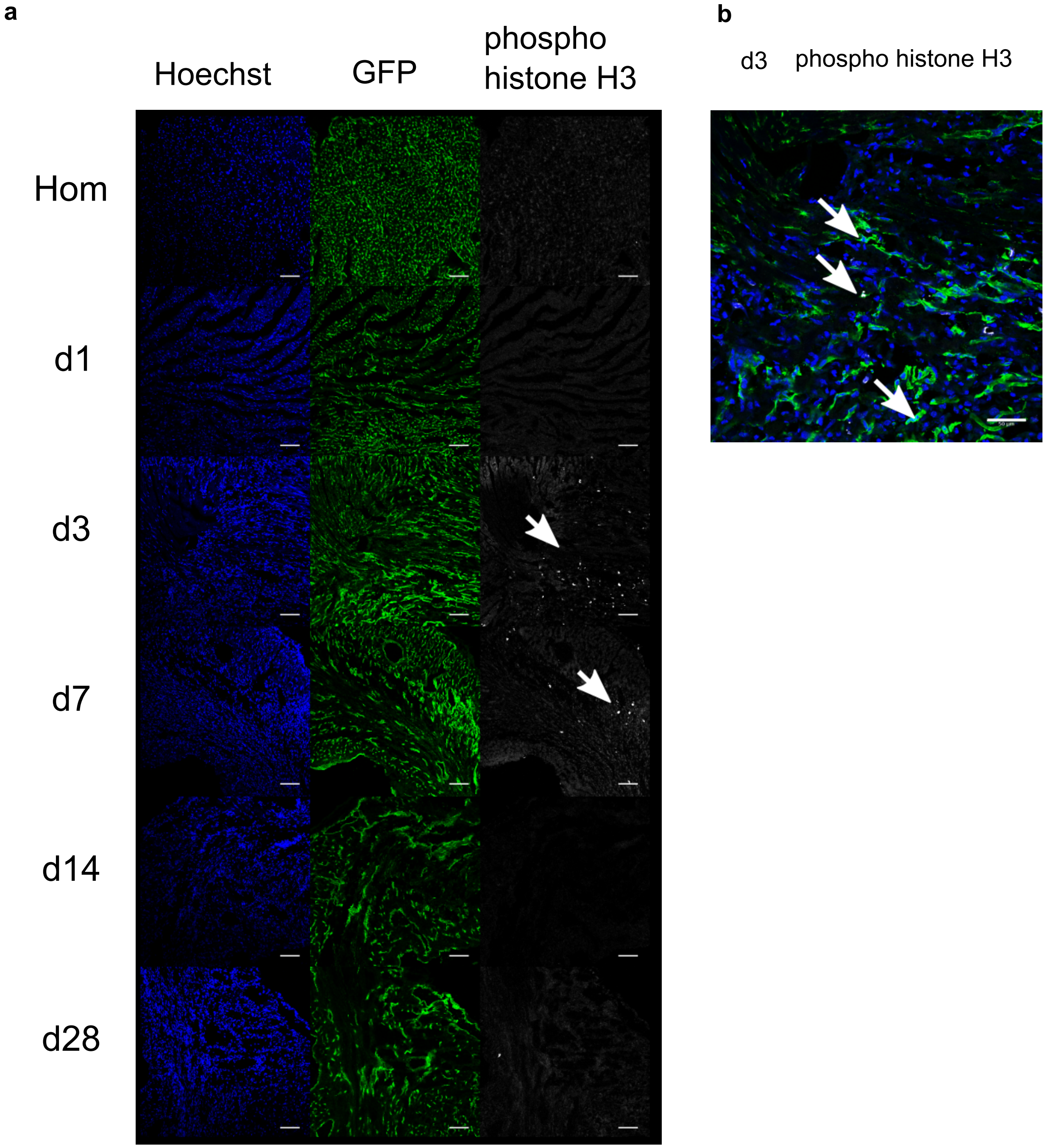

Supplementary Figure 2 – Cell proliferation is increased 3 days after infarction

a Representative images of infarct border zones of tamoxifen treated *Cdh5-CreERT2;mT/mG* mice at the respective time points. Nuclei were stained with Hoechst (blue), endothelial cells were traced by GFP (green) and proliferation was assessed by phospho histone H3 (white) (Hom (homeostasis), days (d) d1, d3, d7, d14, d28 after infarction). Cells at d3 show the highest number of phospho histone H3 positive cells. Arrows indicate regions with high number of phospho histone H3 positive cells. Scale bars indicate 100 µm. **b** Representative higher magnification image of d3 showing proliferating endothelial cells. Arrows mark GFP and phospho histone H3 double positive cells. Representative images of n = 3 per time point are shown. Scale bars indicate 50 µm.

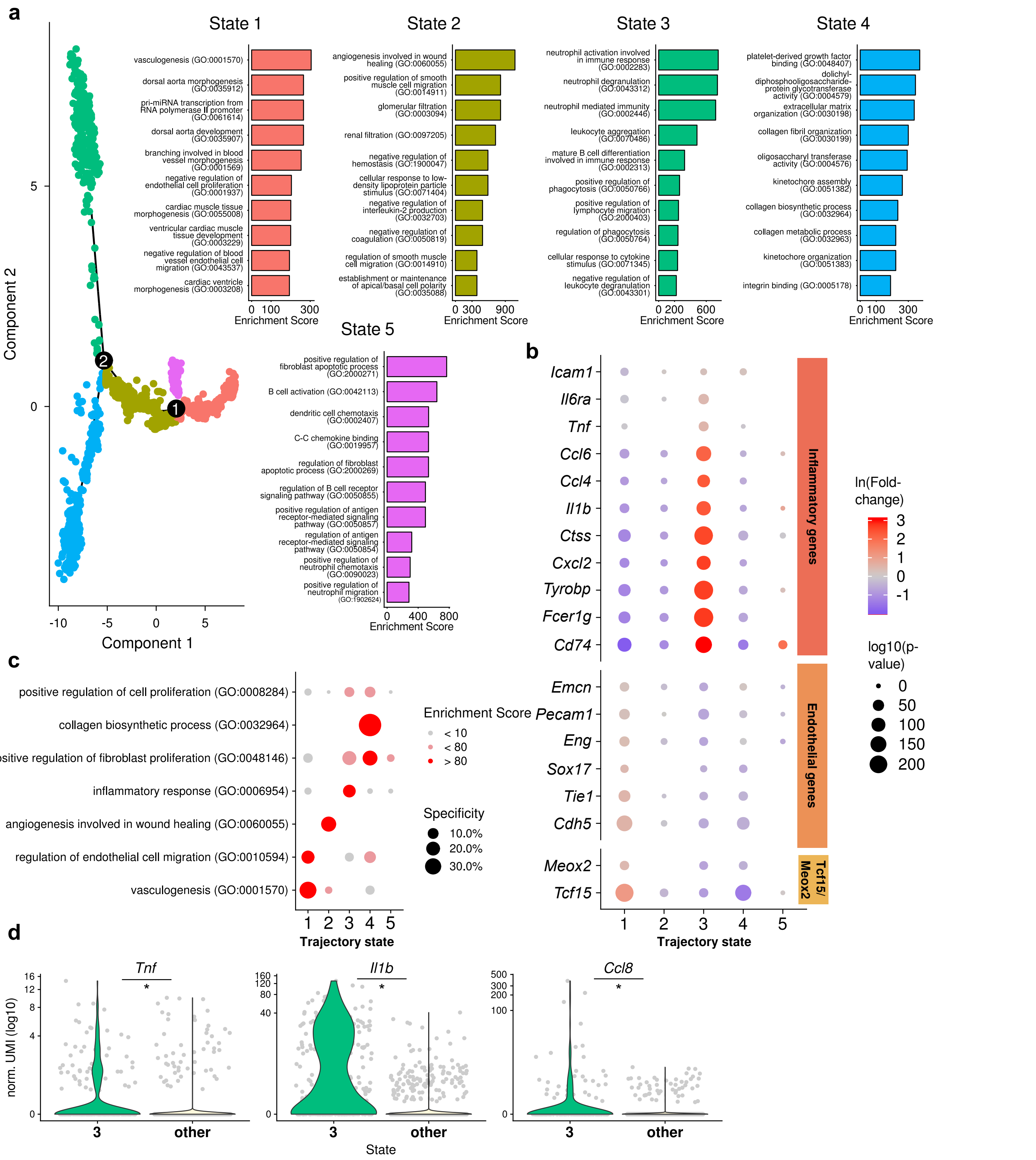

Supplementary Figure 3 – Endothelial states after myocardial infarction

a Pseudo time analysis showing 5 states from Figure 2a. Respectively, top 10 enriched GO-Terms, based on Enrichr's combined score are shown for each state. **b** Inflammatory genes, endothelial genes and transcription factor genes are shown in log fold-change (blue to red) across trajectory states. Significance levels are indicated by size. **c** Enrichment of characteristic GO-Terms defining endothelial states. Enrichment score (combined score) is indicated by color. Size indicates number of significantly regulated genes per total genes in GO-Term. **d** Violin plots showing individual inflammatory markers shown in comparison between state 3 (n = 220 cells) and all other states (n = 2685 cells) p = 2.9 * 10^{-33} (*Tnf*), p = 3.6 * 10^{-110} (*Il1b*), p = 2.9 * 10^{-26} (*Ccl8*), bimodial likelihood-ratio test, Bonferroni adjusted p-values.

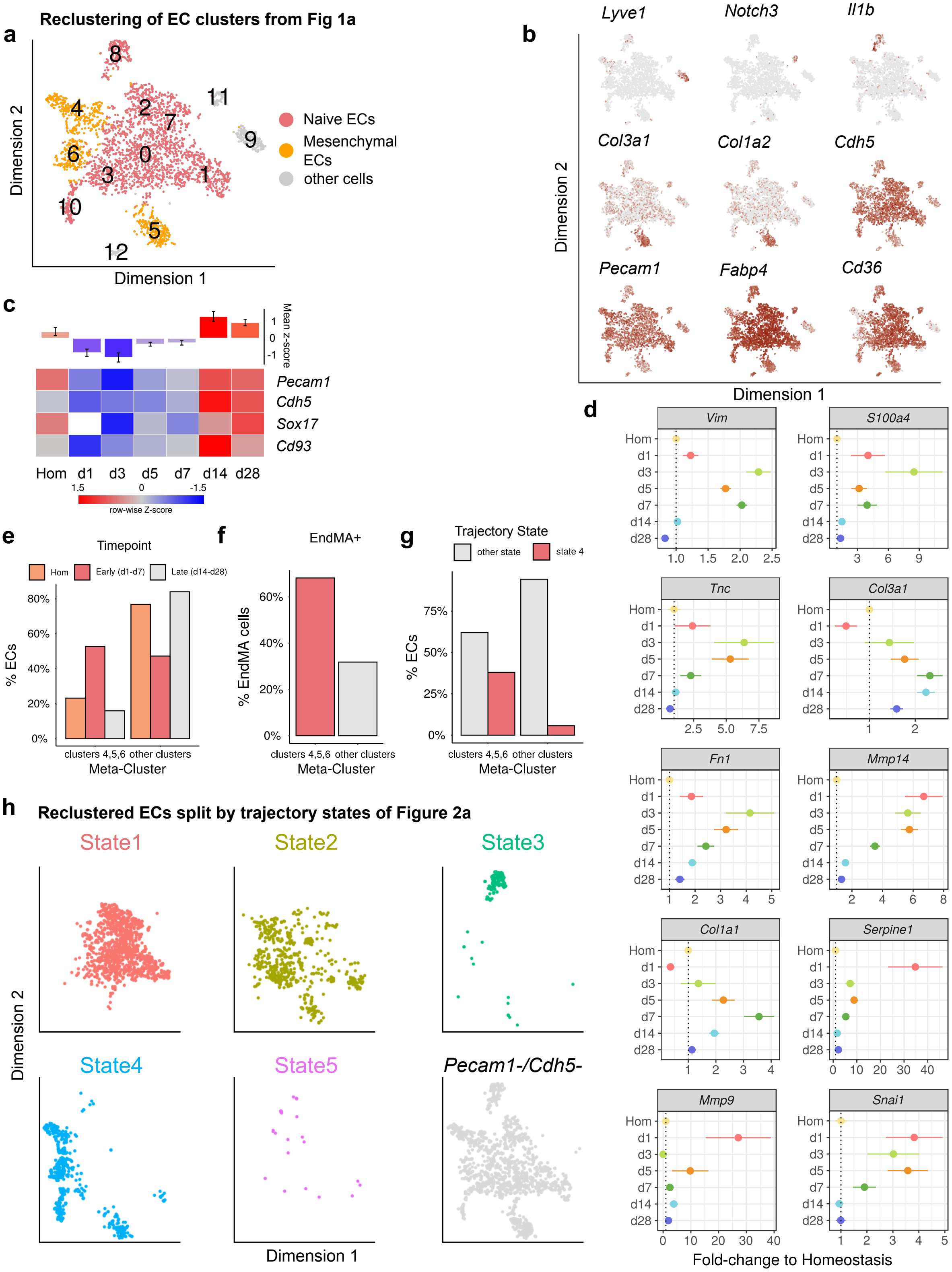

Supplementary Figure 4 – Re-clustering of endothelial clusters recapitulate EndMA phenotype

a tSNE plot showing the re-clustering of cells from endothelial cell (EC) cluster (Figure 1a, Supplementary Figure 1a). Cells highlighted in red represent baseline EC population, orange (4,5,6) indicates cluster with ECs expressing mesenchymal genes, grey indicate cells expressing lymphatic EC (9), pericyte (11) or B-cell (12) markers. **b** tSNE displaying lymphatic EC marker (*Lyve1*) in cluster 9, Pericyte marker (*Notch3*) , inflammatory ECs (*Il1b*), mesenchymal ECs (*Col3a1*, *Col1a2*) and canonical EC markers (*Cdh5, Pecam1, Fabp4, Cd36*). **c** Heatmap showing loss of EC markers in cells from EC cluster in (a) (red, 1-8, 10; cells from grey clusters excluded). Error bars represent SEM. Homeostasis n = 1190 cells, d1 n = 97 cells, d3 n = 74 cells, d5 n = 255 cells, d7 n = 380 cells, d14 n = 992 cells, d28 n = 902 cells. **d** Dot plots showing fold increase of gene expression of mesenchymal marker in ECs cluster as shown in (a) compared to homeostasis (cells from grey clusters excluded). **e-g** Bar plots showing relative contribution to different timepoints (**e**), EndMA cells used in Fig. 3a (**f**) and State 4 cells from trajectory analysis shown in Fig. 2a (**g**) of the mesenchymal clusters 4,5,6 (n = 1003 cells) compared to other EC clusters (0-3,7,8,10, cells from grey clusters excluded, n = 2887 cells). Data represents n = 1 mouse per timepoint. **h** tSNE plot showing re-clustered cells (a), separated by their trajectory state as shown in Figure 2a.

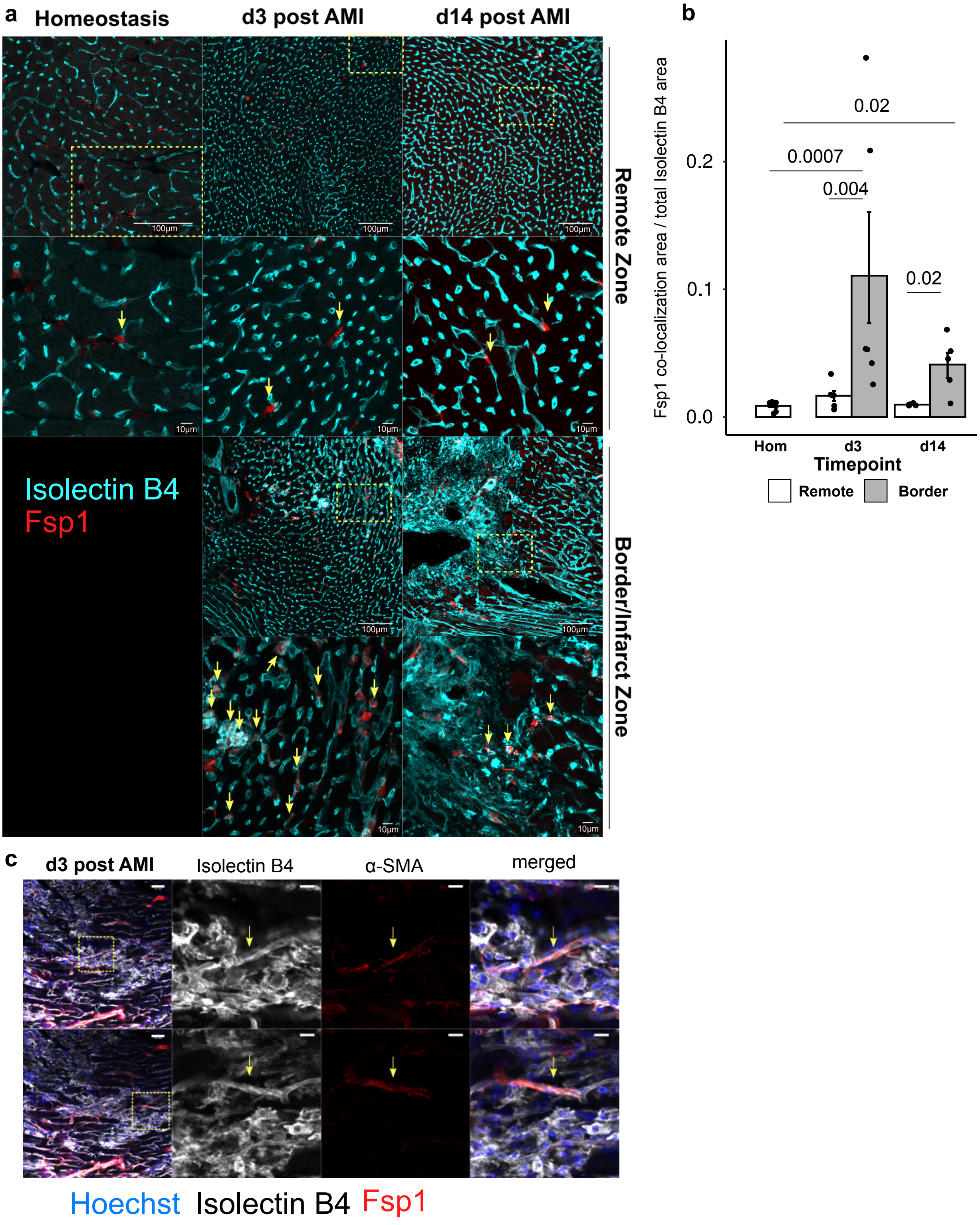

Supplementary Figure 5 – Histological evaluation of EndMA in mice

a Immunostaining of heart cross sections in homeostasis and after MI. Confocal images show remote zone (Homeostasis, d3 post AMI and d14 post MI) and border/infarct zone (d3 post AMI and d14 post AMI). Mesenchymal marker Fsp1 (red) colocalizes with the endothelial marker Isolectin B4 (cyan) in cells close to the infarcted region (yellow arrows). Scale bars 100 µm or 10 µm as indicated. n = 5 mice per group. 6 images of different sections were analysed per animal. **b** Quantification of Fsp1 colocalization with IB4 area normalized to total IB4 area. Data shown as mean ± SEM. P-values were calculated using two-sided Wilcoxon Ranked Sum Test, Homeostasis n = 8, d3 n = 6, d14 n = 5 mice. **c** Representative images of d3 after MI. Cells expressing the mesenchymal marker alpha smooth muscle actin (α-SMA) (red) co-localized endothelial marker Isolectin B4 (white). Images on the left panel represent overview of border zones, right panels represent higher magnification of cells expressing Isolectin B4 and alpha α-SMA and merged images. Scale bars indicates 50 µm (left) or 10 µm (higher magnifications).

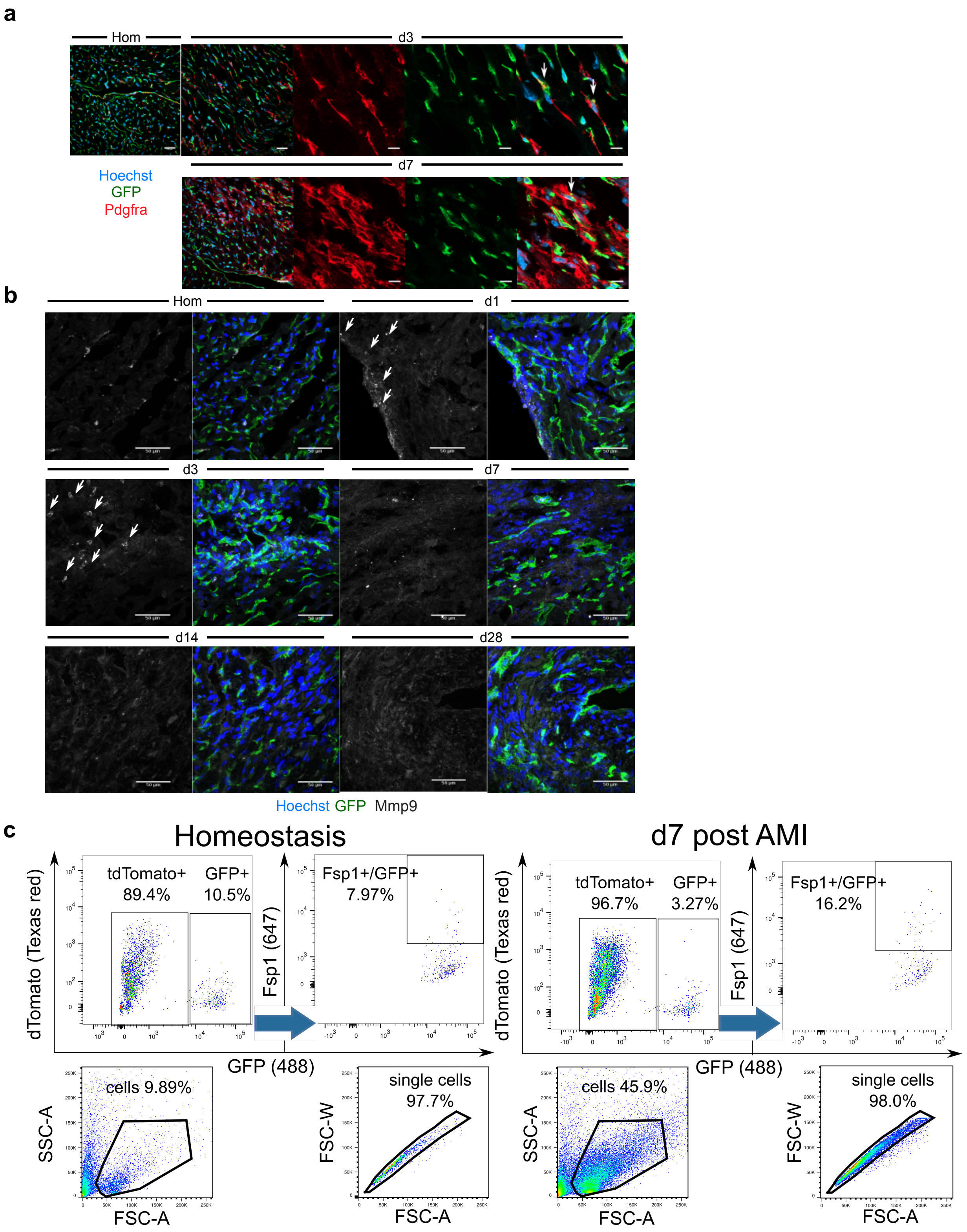

Supplementary Figure 6 – Evaluation of EndMA

a Representative images showing homeostasis and d3 or d7 border zones after myocardial infarction (MI) in *Cdh5-CreERT2;mT/mG* mice. Mesenchymal marker Pdgfra (red) co-localizes with GFP (green) (see arrows). Panels show merged and individual channels of selected regions. Scale bars indicate 30 µm (left) or 10 µm (higher magnifications). **b** Representative images showing time course after MI (homeostasis, d1, d3, d7, d14, d28). Border zones show increased expression of Mmp9 (white) at d1-d7 after infarct (see arrows). Scale bars indicate 50 µm. **c** FACS analysis of homeostasis and d7 cells from the total heart (top panel) GFP^+ cells positive for mesenchymal marker Fsp1(*S100a4*) were increased from homeostasis (7.97% of GFP^+) to d7 (16.2% of GFP^+). Respective gating strategy is shown in the bottom panel.

a

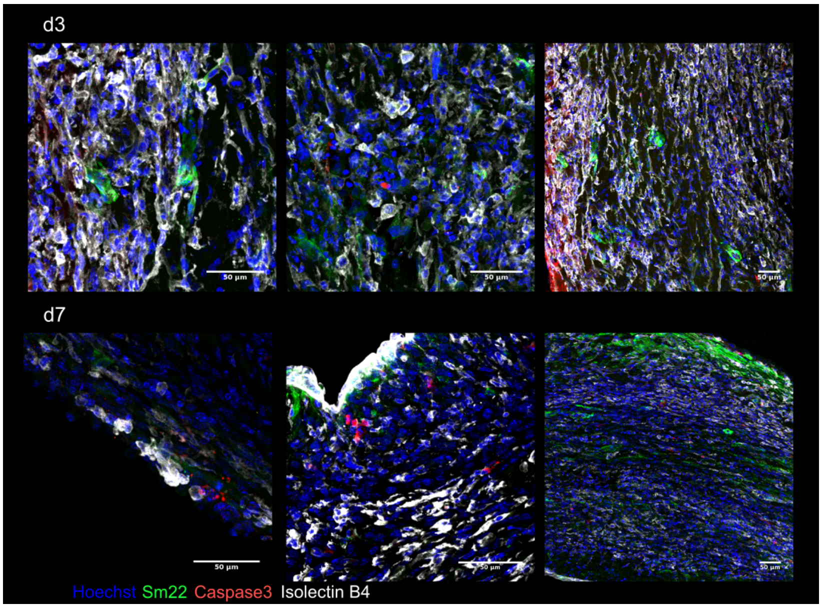

b

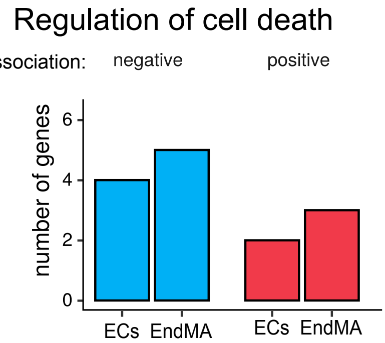

Supplementary Figure 7 - EndMA cells and apoptosis marks

a Immunohistochemistry of border zones at d3 or d7 stained for Hoechst (blue), mesenchymal marker Sm22 (green), apoptosis marker Caspase3 (red) and endothelial marker Isolectin B4 (white). Scale bars represent 50 µm. **b** GO-Term enrichment of genes differential regulated between EndMA+ and EndMA- cells (Figure 3a). Genes were classified by their association to regulate cell death: positive regulation (GO:0010942) or negative regulation (GO:0060548).

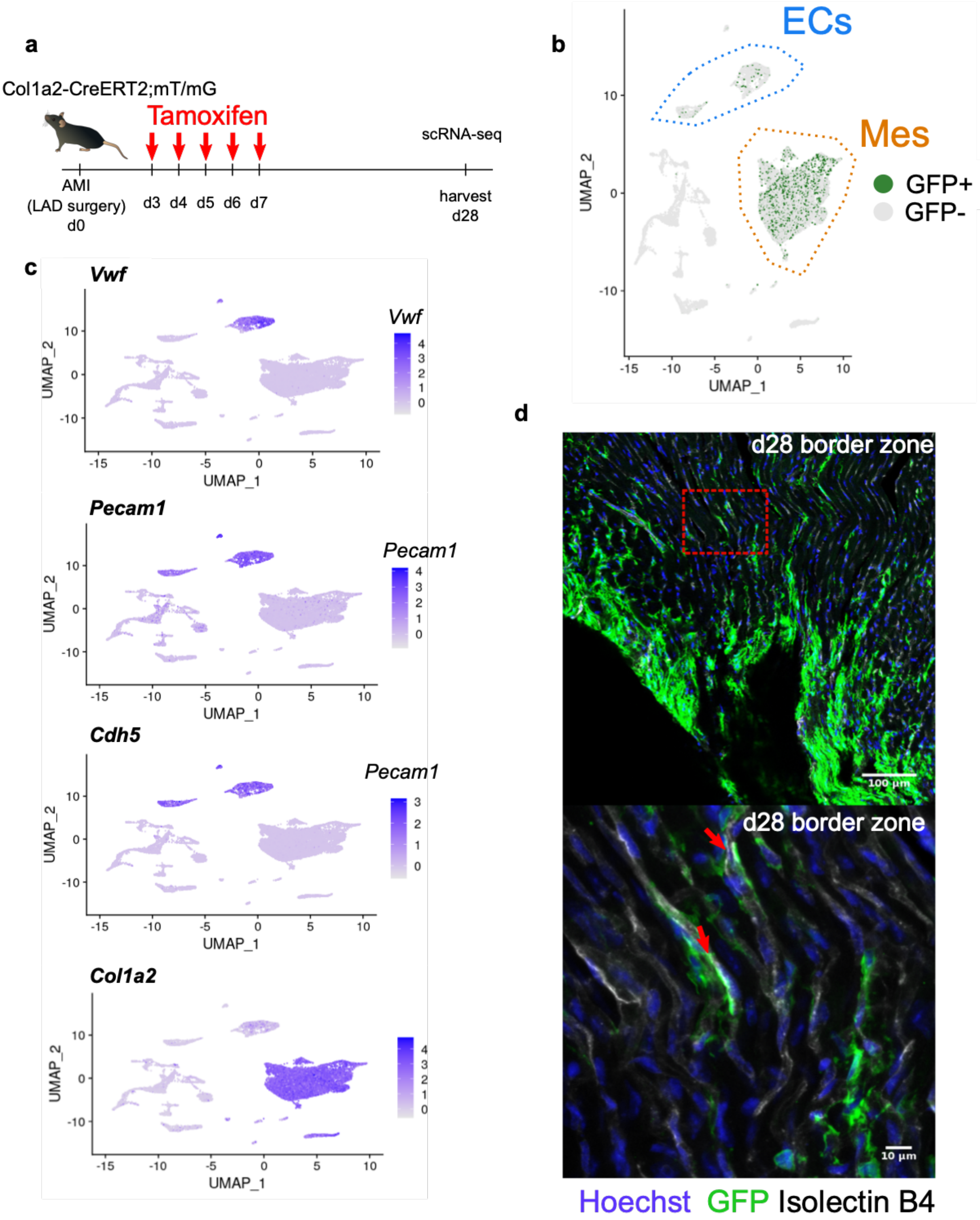

Supplementary Figure 8 - Col1a2-CreERT2 tracing confirms presents of endothelial cells that underwent EndMA after d28

a Schematic illustration of experimental design. We used tamoxifen inducible *Col1a2-CreERT2;mT/mG* adult (12-weeks, *n* = 1) and old (18 months, n = 1) mice to trace cells that express mesenchymal marker *Col1a2* after MI. Tamoxifen was injected daily from d3-d7 after MI. Hearts were collected at d28 after infarct and processed for single cell RNA sequencing or histology. Age-matched littermates (n = 1 each) without LAD surgery were used as control. **b** tSNE plot showing GFP positive cells (green). GFP positive cells were predominantly found in mesenchymal (Mes) clusters, but also in endothelial (EC) clusters. **c** tSNE plot showing different endothelial markers (*Vwf, Pecam1, Cdh5*) and mesenchymal marker *Col1a2* used for determining endothelial and mesenchymal clusters. **d** Border zone of d28 after MI of *Col1a2-CreRT;mT/mG* hearts. Arrows indicate where green signal (GFP) overlaps with white signal (endothelial marker isolectin B4). Bottom panel represents a zoom of selected area. Scale bars represent 100 µm or 10 µm.

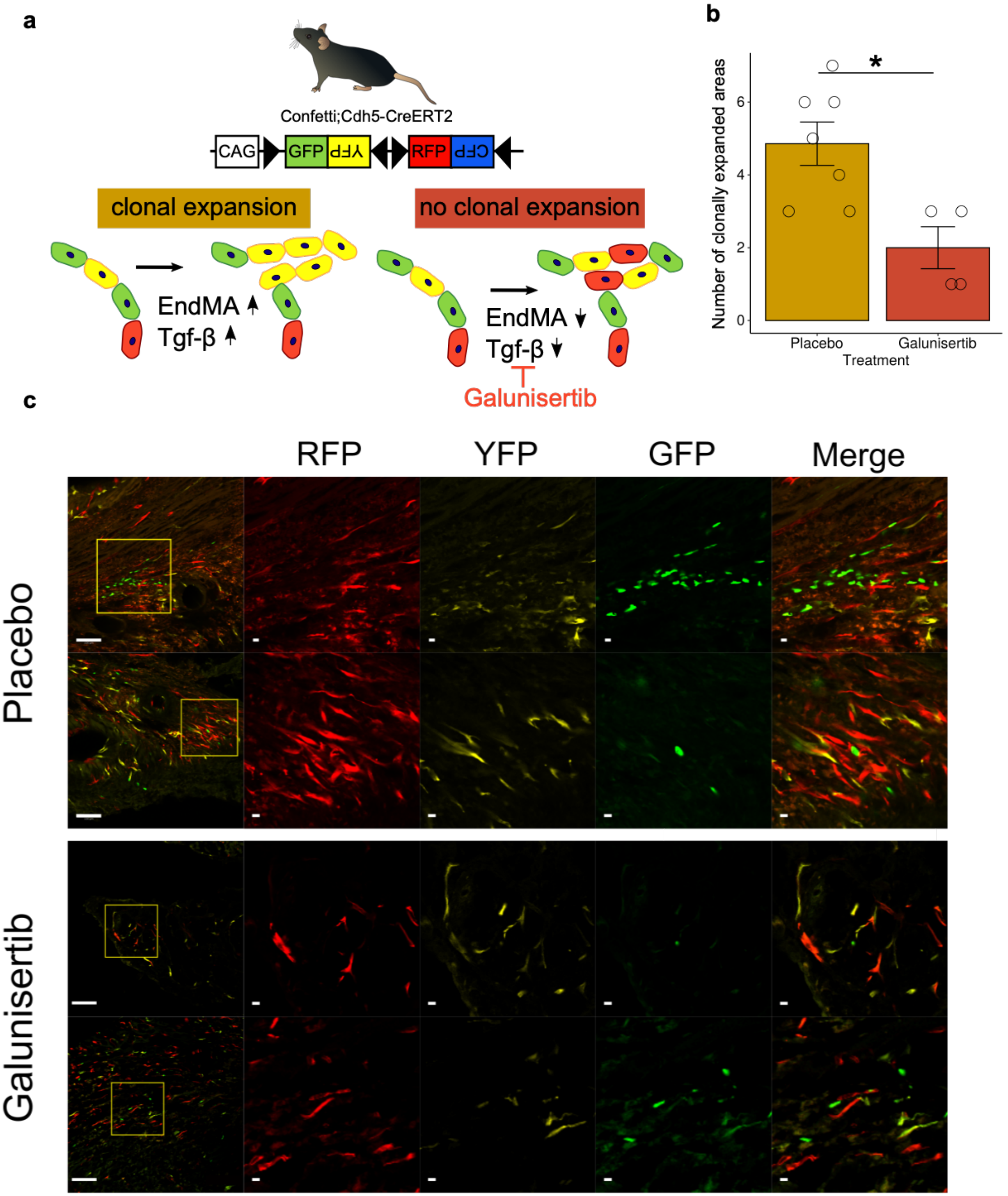

Supplementary Figure 9 – Tgf-β inhibition impairs clonal expansion *in vivo*

a Experimental design of the study. Treatment of *Confetti;Cdh5-CreERT2* mice with tamoxifen induces random recombination of the Confetti locus, leading to expression of one specific colour (CFP, RFP, YFP, GFP) in each cell. CFP was neglected because it was poorly detectable. Animals were treated with Tgf-β inhibitor Galunisertib (75mg/kg bi daily by gavage) or placebo. Mice were harvested at day 7. **b** Quantification of areas per animal (n = 30 per animal). Bar plot shows number of clonal expanded areas defined by more than two times the standard deviation to the animal mean of counted RFP,YFP or GFP cells per area. Galunisertib treated animals (n = 4) showed a significant reduction in areas with clonal expansion compared to placebo (n = 7). P-value was calculated using Fisher's exact test for count data (p = 0.03). **c** Representative images of Placebo (upper panel) and Galunisertib (bottom panel) border zones 7 days after infarct. Single channel and merged images for selected regions are shown. Arrows pointing to expansion of green cells (upper panel) and red cells (second panel). Scale bars represent 100 µm (overview) or 10 µm (higher magnifications).

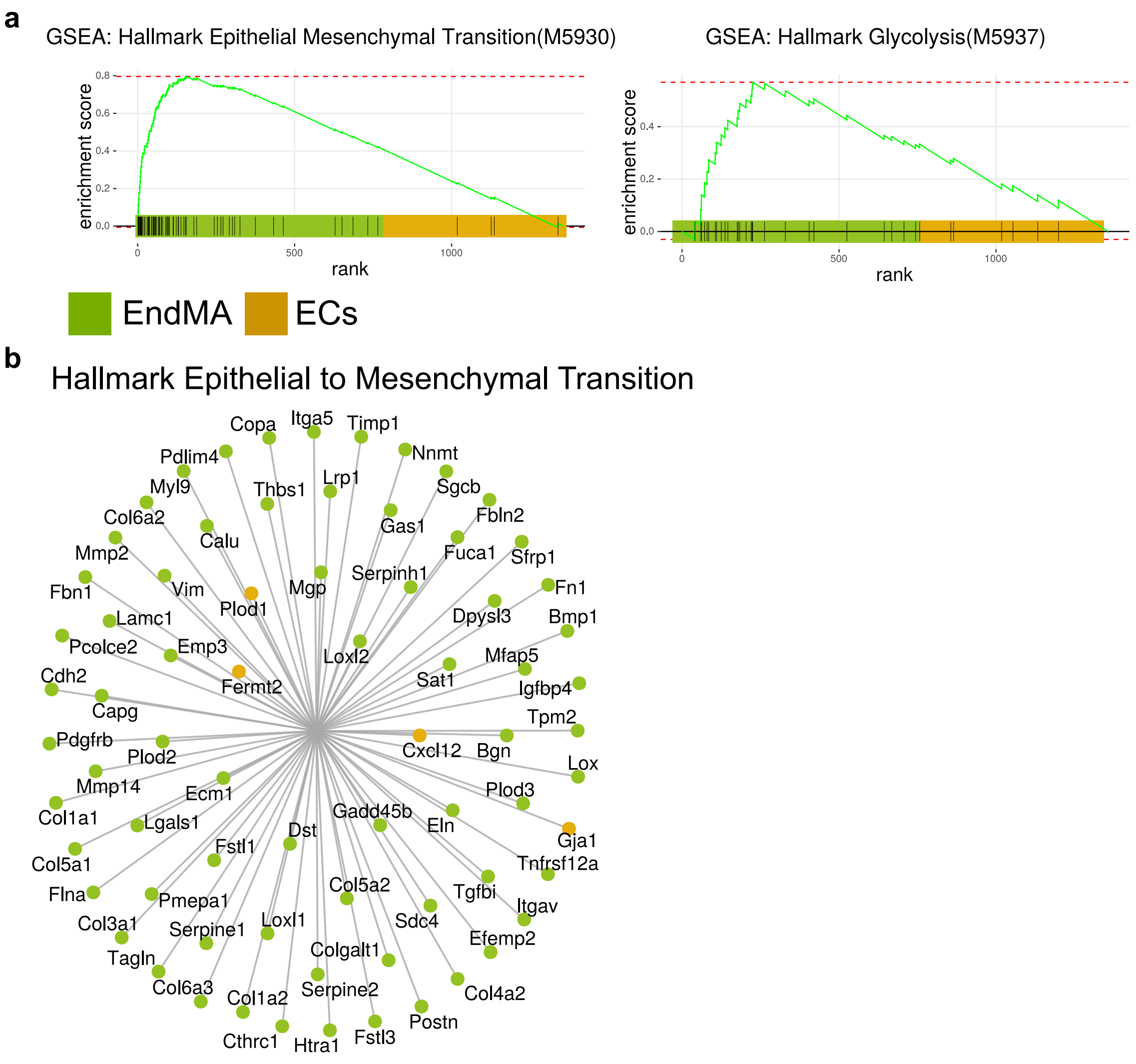

Supplementary Figure 10 – $EndMA^{+}$ cells show hallmarks of epithelial mesenchymal transition and glycolysis

a Gene set enrichment analysis using genes differentially regulated between $EndMA^{+}$ and $EndMA^{-}$ cells, as shown in Figure 3. Genes were enriched against “Hallmark gene sets” taken from Molecular Signatures Database (MSigDB, gsea-msigdb.org). Both gene sets were among top 3 gene sets enriched (Bonferroni adjusted p = 0.002) **b** Genes in term “Hallmark epithelial mesenchymal transition (M5930)” sorted by upregulation either in $EndMA^{+}$ cells or ECs. The majority of genes are upregulated in $EndMA^{+}$ cells (green, n = 64) compared to non-mesenchymal marker expressing ECs (orange, n = 4)

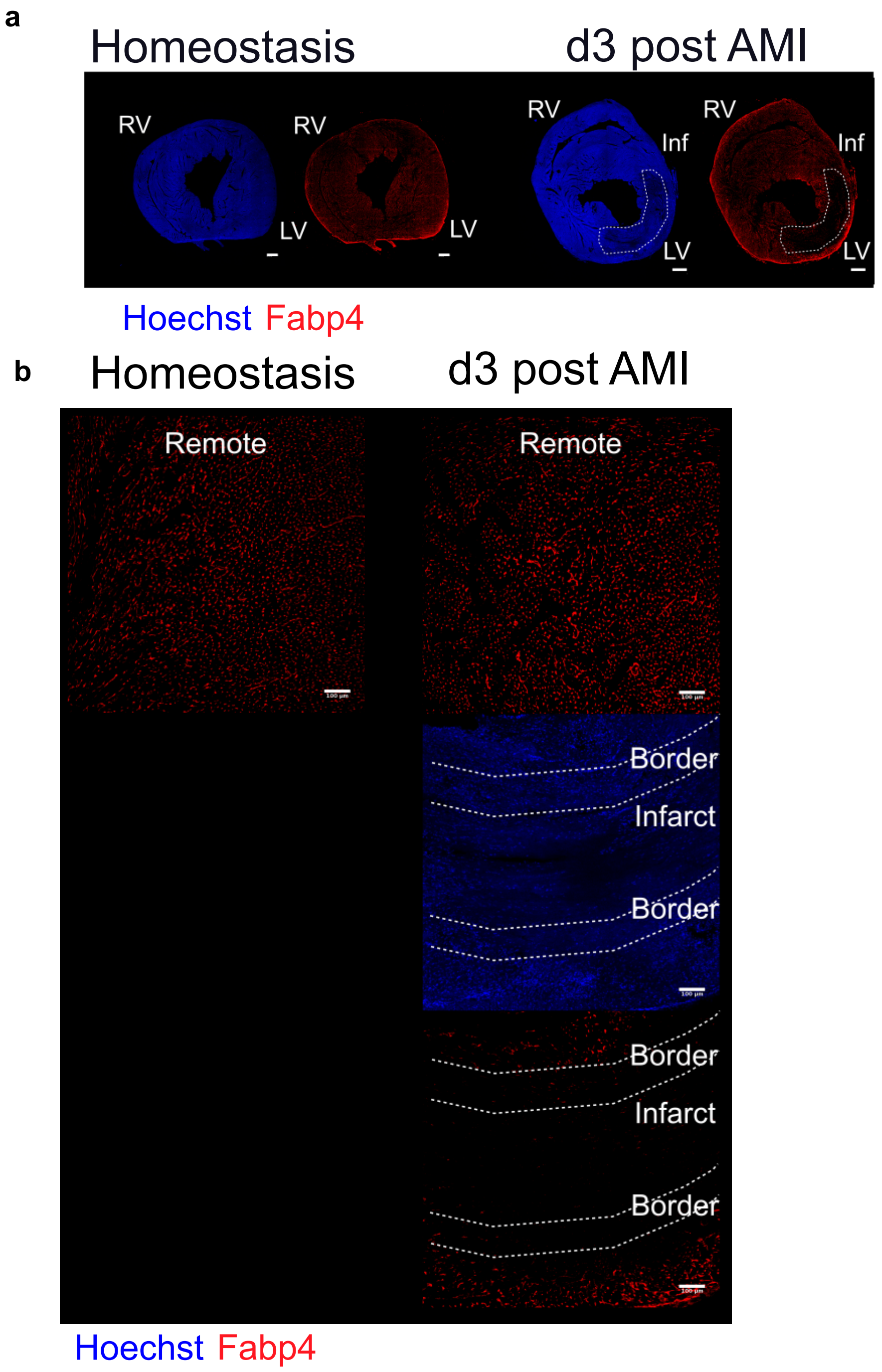

Supplementary Figure 11 – Fabp4 expression is diminished in the border/infarct region

a,b Representative images of heart sections at homeostasis and at day 3 after infarction were, stained with Hoechst (blue) and fatty acid transport protein Fabp4 (red). Cells around the infarct lack the expression of Fabp4. **a** Tile scan of the complete cross section of the heart **b H**igher magnification picture in either remote or border zone regions. A representative image of n = 3 animals analysed is shown. Scale bars indicate 500 µm (tile scan) or 100 µm.

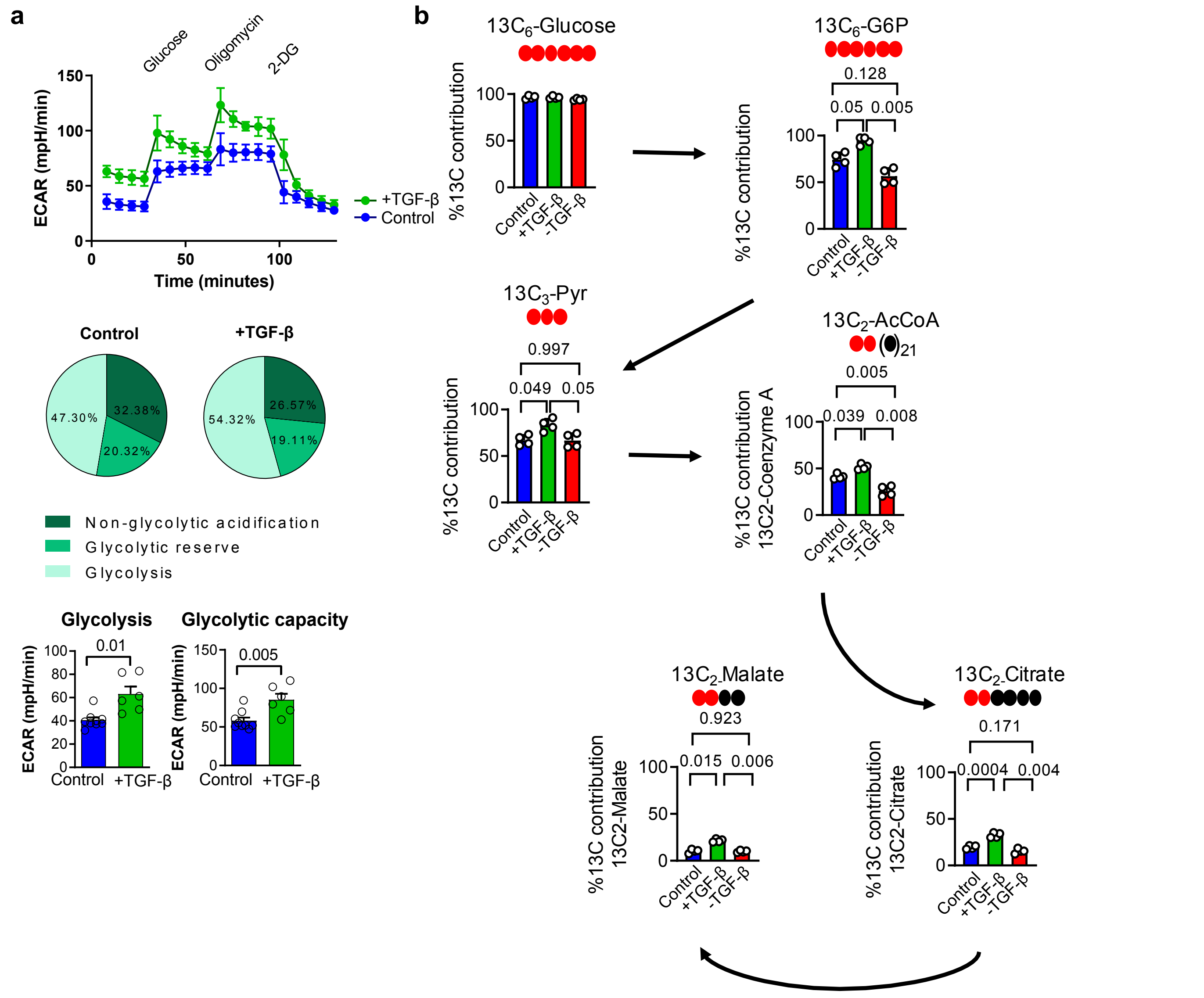

Supplementary Figure 12 – Effects of TGF-β stimulation on glycolysis and glucose metabolism

a HUVECs were treated with TGF-β2 for 7 days to induce EndMA (+ TGF-β2) and glucose metabolism was measured by Seahorse analysis. Data were compared to untreated cells (Control). Glycolytic parameters determined from the extracellular acidification rate measurements, in endothelial cells maintained in the respective media. n = 3/group. 2-DG, 2-deoxy-glucose **b** $^{13}C_6$-Glucose metabolic flux analysis in HUVEC treated with TGF-β2. Cells were either treated with TGF-β2 for 7 days (+ TGF-β2) or 3 days followed by 4 days of withdrawal to control (- TGF-β2). Data compared to untreated cells (Control). Results are presented as %^{13}C contribution to the detected metabolite. n = 4/group. P-value was calculated using One way ANOVA paired test, Bonferroni adjuested. Data shown as mean ± SEM. G6P, Glucose 6Phosphate; Pyr, Pyruvate; AcCoA, Acetyl-Coenzyme A

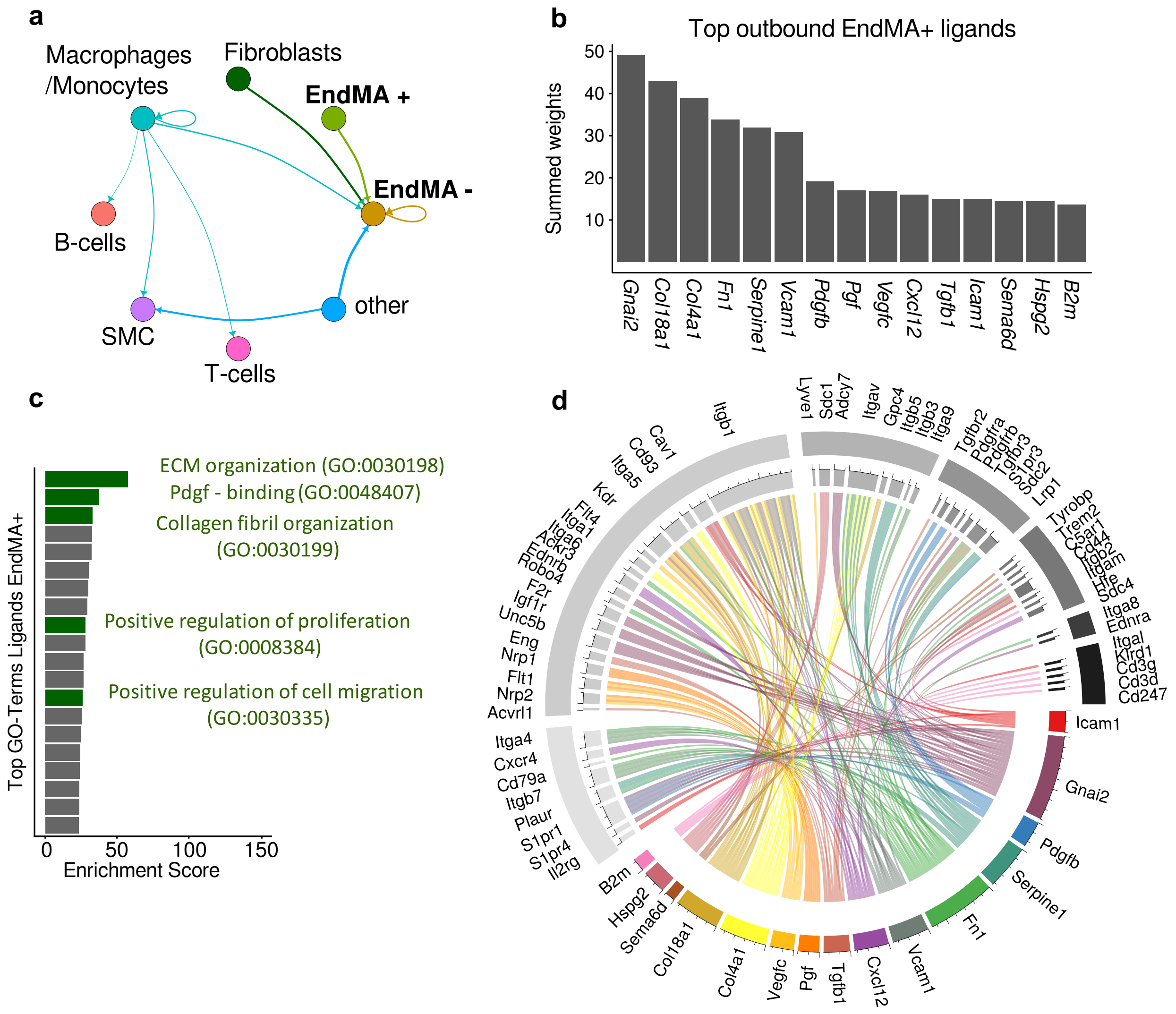

Supplementary Figure 13 – Ligand Receptor interaction prediction shows specific interactions of EndMA+ cells

a Cell-cell communication network of predicted interactions between cell types, EndMA+ and EndMA- endothelial cells. Thickness indicates sum of weighted paths of predicted interactions based on a ligand receptor network. **b** Top 15 outbound ligands for EndMA+ cells measured by weighted analysis on ligand receptor network and their predicted receptors (a). **c** Top 20 GO-terms for ligands predicted in (a), ranked by Enrichr's combined score. **d** Circos plot showing ligand receptor pairs from (b) for top 15 outbound ligands in EndMA+ cells.

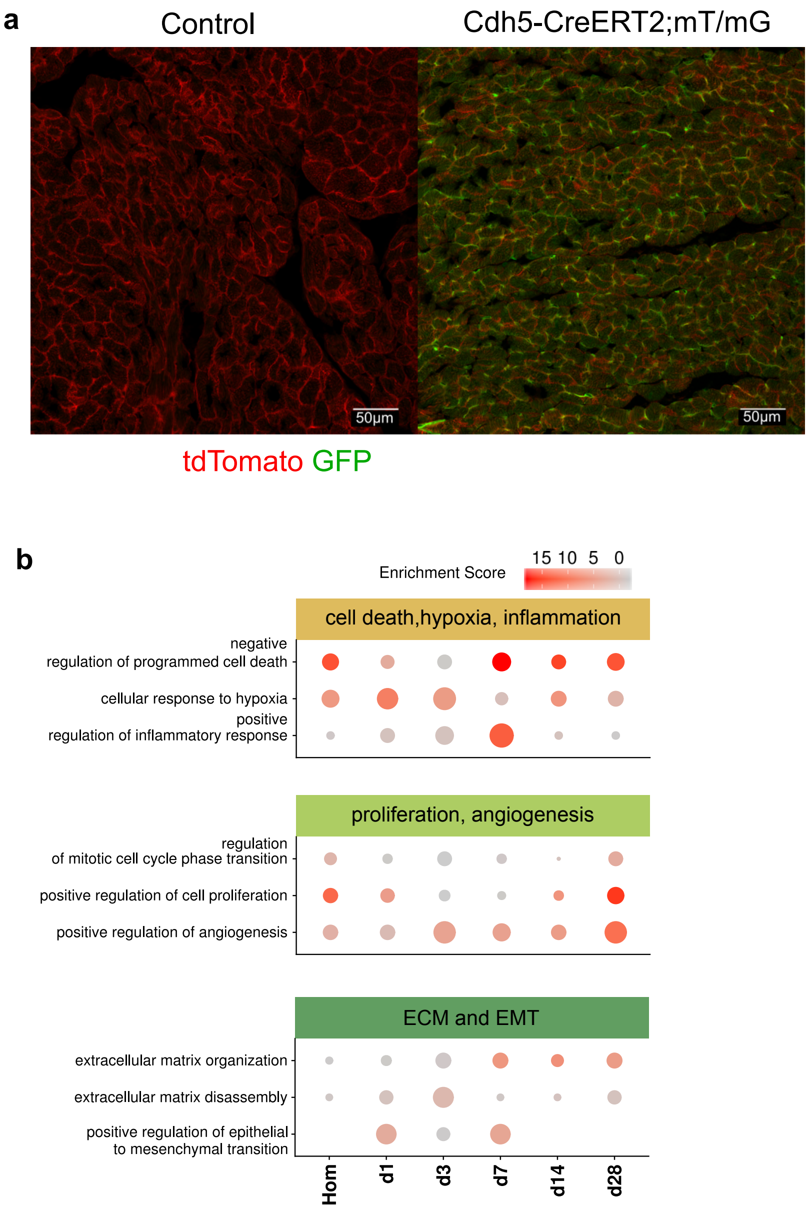

Supplementary Figure 14 – Tracing of EC's reveals pathophysiological changes after MI

a Injection of tamoxifen induces GFP expression in *Cdh5-CreERT2;mT/mG* mice as shown in immunofluorescence images of heart sections **b** GO-terms in GFP expressing cells during the time course after myocardial infarction. GO-Terms are displayed by their combined enrichment score (color) and the relative number of significantly regulated genes enriched per term (dot size).

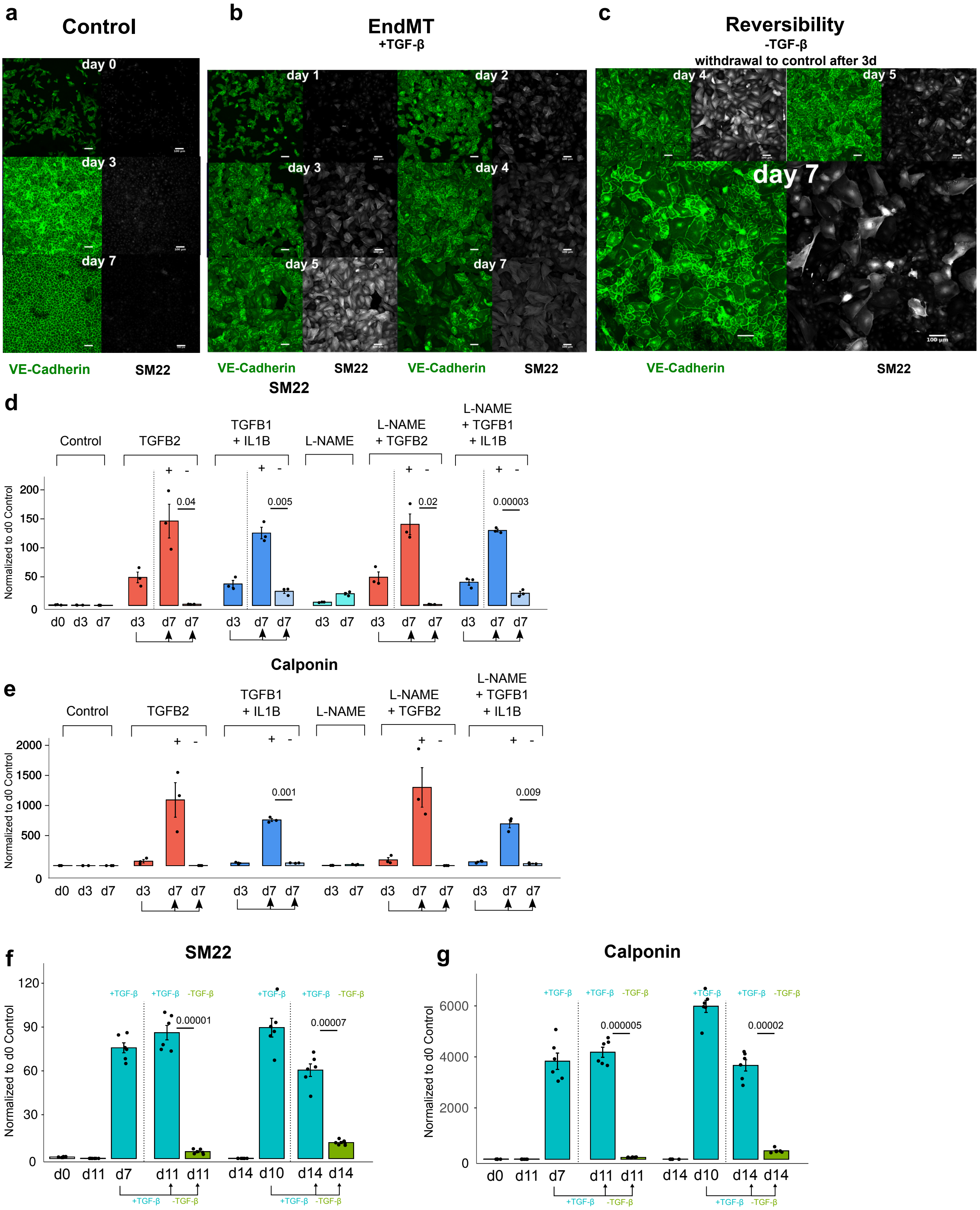

Supplementary Figure 15 – Reversibility of mesenchymal transition in HUVEC cells

a-c Representative images corresponding to Figure 4h and i showing VE-Cadherin and SM22 stainings in HUVECs treated with basal medium (Control) (**a**), treatment of cells with TGF-β2 for the respective time points (**b**), or TGF-β2 treatment for 3 days followed by withdrawal to control medium for the indicated time points (**c**). Scale bars indicate 100 µm. Induction and reversibility is shown at the indicated time points after initiation of treatment (day(d) 1, d2, d3, d4, d5, d7). **d-e** Fold change of *SM22* (**d**) and *Calponin* (**e**) mRNA levels in HUVECs under different EndMT-promoting conditions. Cells have been treated, either with TGF-β2 (red) or 10 ng/mL TGF-β1 + 10 ng/mL IL1B (blue). Addition of eNOS inhibitor 1 mM L-NAME did not change the observed phenotype. Data is normalized to d0 control. n = 3 per group per time point. P-value was calculated using two-sided Student's *t*-test. Data shown as mean ± SEM. **f-g** Fold change of *TAGLN* (SM22) (**f**) and Calponin (*CNN1*) (**g**) of mRNA levels in HUVEC after TGF-β2 treatment for 7d or 10d followed by extended TGF-β2 treatment (blue) or removal of TGF-β2 and switch to basal medium (green) up to d11 or d14. Data is normalized to d0 control. HUVECs cultured in basic conditions for d11 or d14 showed no change to d0. n = 6 per group per time point. P-value was calculated using two-sided Student's *t*-test. Data shown as mean ± SEM

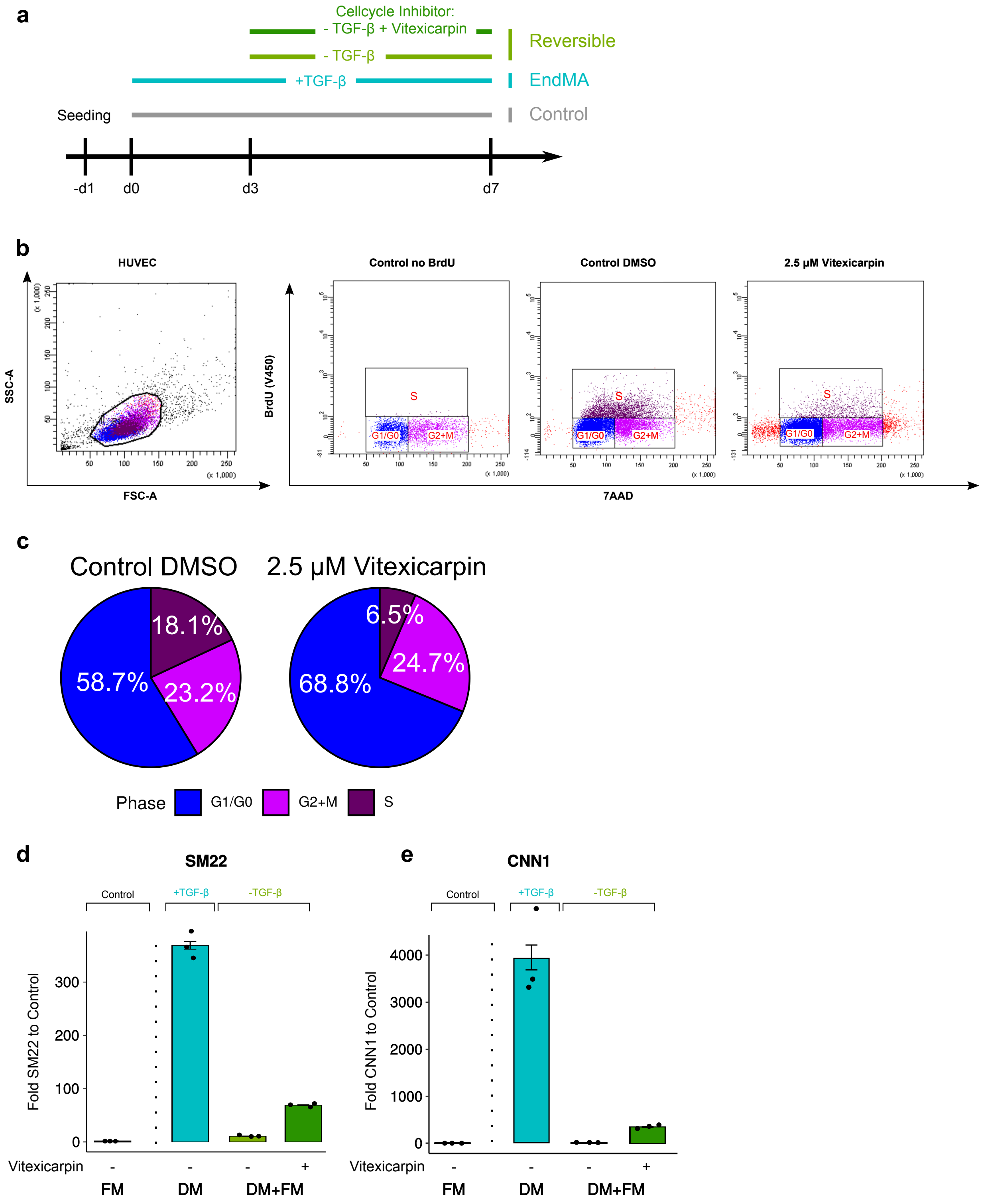

Supplementary Figure 16 - Cellcycle inhibition of HUVEC treated with TGF-β2 does not change reversibility

a Overview of experimental design. HUVEC were seeded and cultivated for 24h in control conditions. We treated the cells with TGF-β2 (see methods sections) and changed the differentiation medium to control medium after 3 days. Cells were then either treated with 2.5 µM Vitexicarpin or DMSO as control (Reversible). We used cells cultivated for 7 days in control medium (Control) and cells cultivated for 7 days in differentiation medium (EndMA) supplemented with TGF-β2 as controls. **b** BrdU assay of HUVECs cultivated in control medium and supplemented with 2.5 µM Vitexicarpin (right panel) or DMSO as control (middle panel) for 24h (n = 2 independent experiments). Cells, which were not treated with BrdU were used as a negative control for defining the gates (left panels). **c** Quantification of BrdU FACS assay shown in **b**. Pie chart represent the mean relative number of cells found in the G1/G0-phase (blue), G2/M-phase (pink) or S-phase (purple). Vitexicarpin treatment reduced the number of cells in S-phase by 63.89% ± 4.79% compared to DMSO control. **d-e** qPCR showing reduction of *TAGLN* (SM22) (**d**) and *CNN1* (**e**) levels after withdrawal of differentiation medium. Data was normalized to control and housekeeping genes *GAPDH* and *RPLP0* and shown as fold-change. Error bars represent standard error of the mean of replicates of n = 3 experiments. Cells in reversible + DMSO had a significant decrease in *TAGLN* (97.10% ± 0.18%, p = 0.002) and *CNN1* (99.5% ± 0.05%, p = 0.02) expression compared to EndMA. Samples with cell cycle inhibitor Vitexicarpin had a similar decrease in *TAGLN* (81.91% ± 0.61%, p = 0.002) and *CNN1* (91.89% ± 0.84%, p = 0.02). Significance was calculated with a two-sided Student's *t*-test.

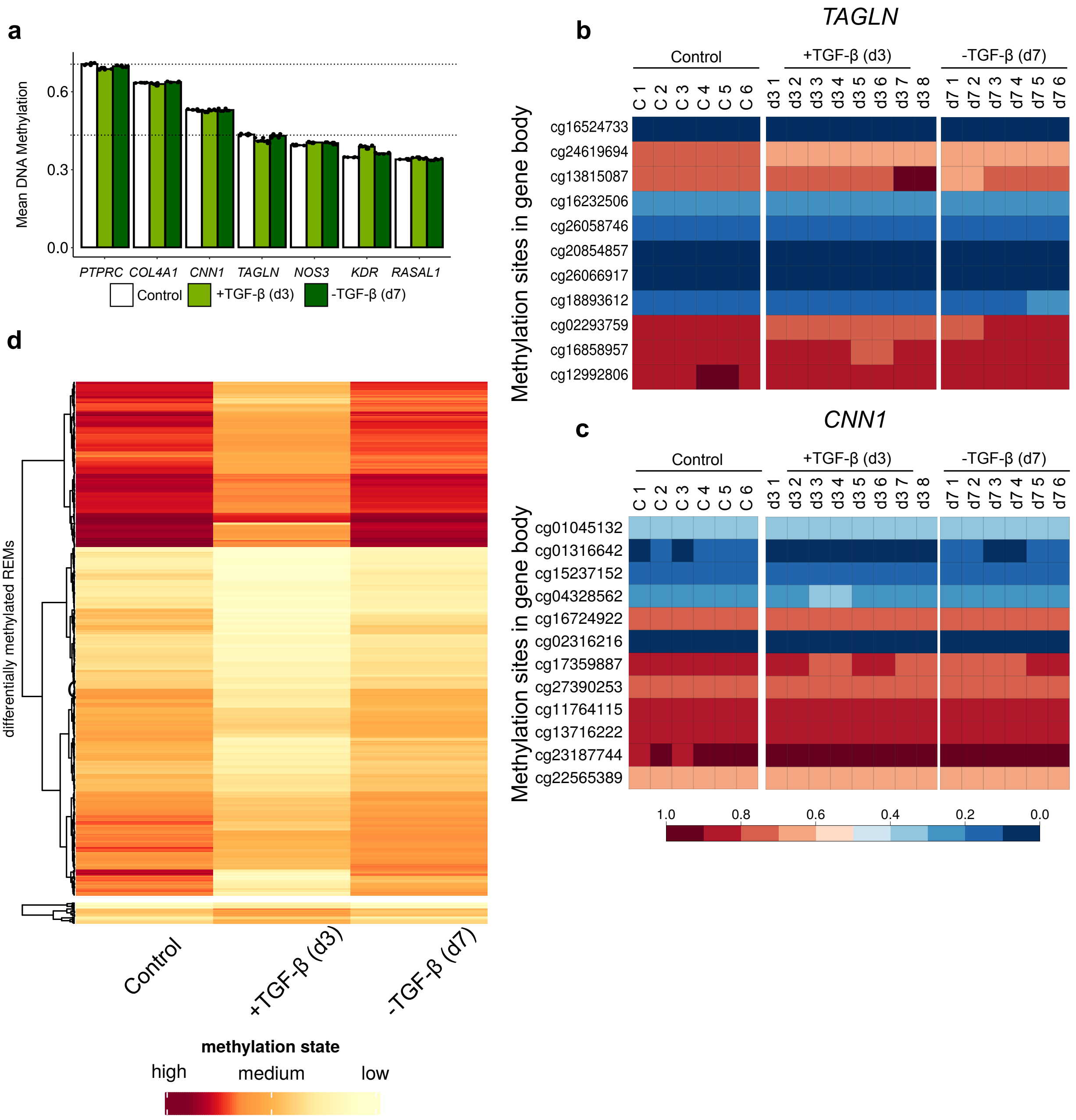

Supplementary Figure 17 – Effect of TGF-β2 on DNA methylation *in vitro*

a DNA methylation was assessed on HUVECs cultured in control conditions (n = 6) for 3 days, TGF-β2 supplemented differentiation medium for 3 days (+TGF-β2 (d3) (n = 8). At day 3, medium was replaced by normal basal complete medium for 4 days (-TGF-β2 (d7) (n = 6). Bar plot shows mean methylation across measured sites (gene bodies) for selected genes associated with non-endothelial identity (*PTRPC, COL4A1*), genes highly regulated with TGF-β2 treatment (*CNN1,* SM22(*TAGLN*)) and representative endothelial marker genes (*NOS3,* VEGFR2 (*KDR*)). **b** Heatmap of methylation sites in the gene body for *TAGLN*. **c** Heatmap of methylation sites in the gene body of *CNN1*. **d** Heatmap showing mean DNA methylation of regulatory elements (REMs). Differential methylated regions with a mean methylation difference ≥ 0.1 between Control and +TGF-β2 (d3) or +TGF-β2 (d3) and -TGF-β2 (d7) respectively. FDR corrected p-value ≤ 0.05 were selected. REMs were clustered by their DNA methylation pattern using hierarchical clustering. Among these differentially regulated REMs, the majority (96%, upper part of the heatmap) followed a pattern of less methylation in +TGF-β2 (d3) but regained methylation levels to baseline conditions in -TGF-β2 (d7)

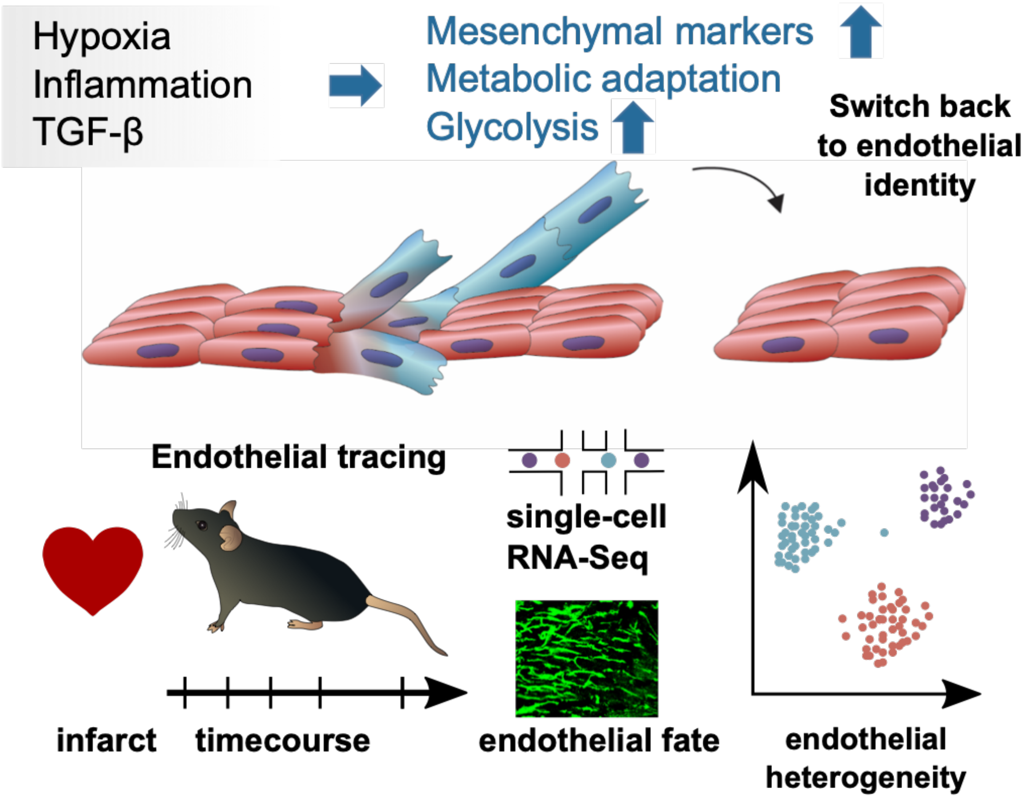

Supplementary Figure 18 – Schematic of study layout

In this study we highlight the phenotypic changes that endothelial cells undergo after myocardial infarct. Upon injury, the microenvironment in the infarcted heart changes, leading to hypoxia, inflammation and increased presence of Tgf-β. These factors induce a plastic and transient expression of a mesenchymal gene program together with changes in the metabolism of the endothelial cells such as increased glycolysis. *In vivo*, single cell RNA sequencing and GFP- lineage tracing of endothelial cells in *Cdh5-CreERT2;mT/mG* mice over time suggest that endothelial cells at later timepoints (d14-d28) transiently switch back to their baseline endothelial identity. This endothelial-to-mesenchymal activation may facilitate endothelial cell migration and clonal expansion crucial to repair the vascular network after cardiac injury.

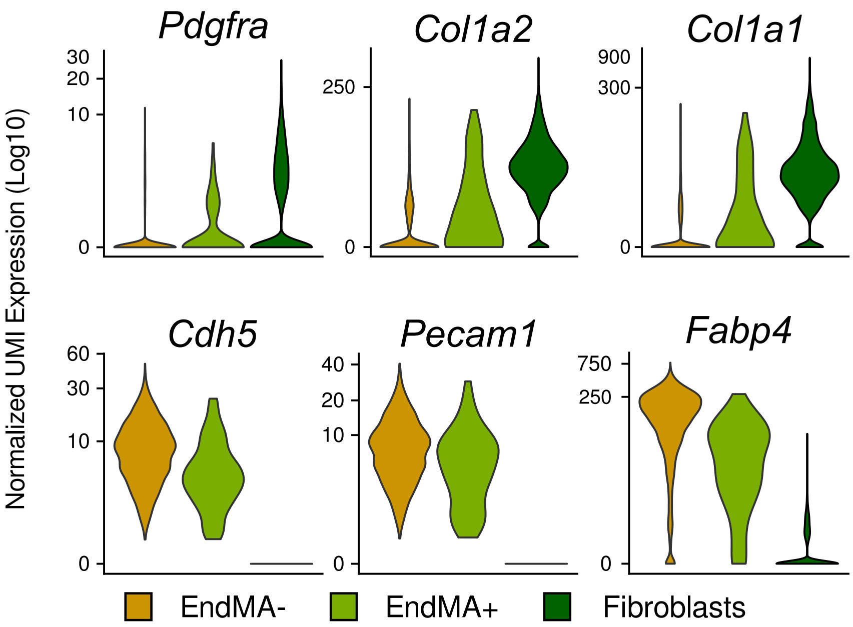

Supplementary Figure 19 – Expression of endothelial and mesenchymal markers in EndMA+ cells

Violin plots showing normalized UMI expression levels of mesenchymal markers (*Pdgfra*, *Col1a2, Col1a1*) and endothelial markers (*Cdh5, Pecam1, Fabp4*) in EndMA-, EndMA+ cells and fibroblasts (defined by clusters in Supplementary Figure 1a).

Single cell RNA-Seq AMI Timecourse (Raw Data *Forte et al.*)									
**Sample**	**Cell Ranger Version**	**Estimated Cells Cellranger**	**Median UMI per cell**	**Mean Reads per cell**	**Median Genes per cell**	**Sequencing saturation**	**Fraction Reads in cells**	**% cells filtered after Quality control**	**% genes filtered after Quality control**
Homeostasis	2.1.1	6,882	2,368	17,660	1,132	71.3%	78.6%	4.56%	19.1%
d1 post	2.1.1	3,527	4,610	39,194	1,751	74.1%	79.2%	2.1%	
d3 post	2.1.1	4,014	4,855	35,806	1,731	68.5%	77.9%	3.76%	
d5 post	2.1.1	5,671	4,072	26,260	1,546	65.8%	77.4%	1.52%	
d7 post	2.1.1	5,928	4,233	28,621	1,610	68.1%	76.8%	1.79%	
d14 post	2.1.1	5,924	4,767	64,728	1,980	83.6%	80.9%	3.88%	
d28 post	2.1.1	4,544	4,557	83,352	1,912	86.2%	77.1%	4.78%	
Mean (±SEM)		5,213 (±456)	4,209 (±324)	42,232 (±8,853)	1,666 (±106)	73.94% (±3.01%)	78.27% (±0.54%)	3.20% (±0.52%)	19.1%

Single cell RNA-Seq lineage tracing Cdh5-CreERT2;mT/mG									
**Sample**	**Cell Ranger Version**	**Estimated Cells Cellranger**	**Median UMI per cell**	**Mean Reads per cell**	**Median Genes per cell**	**Sequencing saturation**	**Fraction Reads in cells**	**% cells filtered after Quality control**	**% genes filtered after Quality control**
Homeostasis	3.0.1	1,791	3,195	68,113	1,314	89.3%	91.5%	14.18%	32.13%
d1 post	3.0.1	1,176	3,787	159,511	1,467	92.7%	91.3%	11.82%	26.78%
d3 post	3.0.1	1,405	5,494	142,018	1,785	89.2%	91.8%	11.34%	21.61%
d7 post	3.0.1	2,096	4,594	102,613	1,685	87.9%	92.2%	9.56%	32.28%
d14 post	3.0.1	2,446	3,064	203,484	1,266	96.3%	92,00%	11.32%	32.09%
d28 post	3.0.1	3,213	3,017	132,482	1,248	95,00%	93.2%	9.84%	32.88%
Tamoxifen Con.	3.0.1	2,630	2,689	191,421	1,152	96.6%	93.1%	12.36%	35.44%
Mean (±SEM)		2,108 (±270)	3,691 (±383)	142,806 (±18,012)	1417 (±90)	92.43% (±1.38%)	92.16% (±0.28%)	11.49% (±0.59%)	30.46% (±1.77%)

Supplementary Table 1 – Single cell RNA-sequencing data

Antigen	Dilution	Catalog number	Source
anti-aSMA-Cy3	1:100	C6198	Sigma
anti-BrdU	1:20	560810	BD Horizon
anti-cleaved Caspase3	1:100	9661	Cell Signaling Technology
anti-CDH5	1:200	2500S	Cell Signaling Technology
anti-Fabp4	1:30	15872-1-AP	Proteintech
anti-Fsp1	1:100 (IHC)<br>1:30 (FACS)	07-2274	Milipore
anti-GFP	1:400 (IHC)<br>1:100 (FACS)	GFP-1010	Aves
anti-GFP Fluorescein Alexa 488	1:50	A11090	Invitrogen
anti-Mmp9	1:300	ab38898	Abcam
anti-Pdgfra	1:100	AF1062	R&D
anti-phoshoH3	1:100	06-570	Milipore
anti-Sm22	1:100	ab10135	Abcam
biotinylated Isolectin B4	1:50	B1205	Vector
anti-goat IgG Alexa 647	1:200	A-21447	Thermo Fisher
anti-rabbit IgG Alexa 647	1:200 (IHC)<br>1:100 (FACS)	A-31573	Thermo Fisher
anti-rat IgG Alexa 647	1:200	ab150155	Abcam
anti-rabbit Alexa 555	1:200	A31572	Thermo Fisher
SAV Alexa 647	1:200	S32357	Invitrogen
anti-chicken IgY Alexa 488	1:200 (IHC)<br>1:100 (FACS)	F-1005	Aves
Hoechst 33342	1:400	AS83218	Ana Spec

Supplementary Table 2 – Antibodies used in this study

Primer names	Sequences (5’->3’)	Target gene	Species
RPLP0-fw	GGCGACCTGGAAGTCCAACT	*RPLP0*	human
RPLP0-rv	CCATCAGCACCACAGCCTTC	*RPLP0*	human
GAPDH-fw	ATGGAAATCCCATCACCATCTT	*GAPDH*	human
GAPDH-rev	CGCCCCACTTGATTTTGG	*GAPDH*	human
SM22-fw	AAGAATGATGGGCACTACCG	*TAGLN*	human
SM22-rv	ATGACATGCTTTCCCTCCTG	*TAGLN*	human
CNN1-fw	CTGGCTGCAGCTTATTGATG	*CNN1*	human
CNN1-rv	CTGAGAGAGTGGATCGAGGG	*CNN1*	human

Supplementary Table 3 – Primers used in this study
