# Supplementary material for: Single cell sequencing reveals endothelial plasticity with transient mesenchymal activation after myocardial infarction
Source: Nat Commun. 2021 Jan 29;12:681. doi: 10.1038/s41467-021-20905-1 (PMC7846794; doi:10.1038/s41467-021-20905-1)
Supplement: Supplementary file 3 — Description of Additional Supplementary Files [file 41467_2021_20905_MOESM3_ESM.docx]

**Supplementary data 1 – Cell annotations as shown in Supplementary Figure 1b**

Table showing cell barcodes obtained from single cell RNA-sequencing AMI timecourse (*Forte et al*.) and their corresponding cluster from this study ‘Cluster Tombor et al.’ and the original clustering ‘Cluster Forte et al.’ as well as the original cell type annotations. Barcodes (n = 30714) were only considered when they have been included in both datasets and not removed by quality control and showed unique identification across libraries. Data is visualised in Supplementary Figure 1b.
