# Supplementary material for: Single cell sequencing reveals endothelial plasticity with transient mesenchymal activation after myocardial infarction
Source: Nat Commun. 2021 Jan 29;12:681. doi: 10.1038/s41467-021-20905-1 (PMC7846794; doi:10.1038/s41467-021-20905-1)
Supplement: Supplementary file 5 — Reporting Summary [file 41467_2021_20905_MOESM5_ESM.pdf]

nature research

Corresponding author(s): Prof. Stefanie Dimmeler

Last updated by author(s): Nov 18, 2020

Reporting Summary

Nature Research wishes to improve the reproducibility of the work that we publish. This form provides structure for consistency and transparency in reporting. For further information on Nature Research policies, see our Editorial Policies and the Editorial Policy Checklist.

Statistics

For all statistical analyses, confirm that the following items are present in the figure legend, table legend, main text, or Methods section.

n/a	Confirmed	
☐	☒	The exact sample size (*n*) for each experimental group/condition, given as a discrete number and unit of measurement
☐	☒	A statement on whether measurements were taken from distinct samples or whether the same sample was measured repeatedly
☐	☒	The statistical test(s) used AND whether they are one- or two-sided<br>*Only common tests should be described solely by name; describe more complex techniques in the Methods section.*
☒	☐	A description of all covariates tested
☐	☒	A description of any assumptions or corrections, such as tests of normality and adjustment for multiple comparisons
☐	☒	A full description of the statistical parameters including central tendency (e.g. means) or other basic estimates (e.g. regression coefficient) AND variation (e.g. standard deviation) or associated estimates of uncertainty (e.g. confidence intervals)
☐	☒	For null hypothesis testing, the test statistic (e.g. *F*, *t*, *r*) with confidence intervals, effect sizes, degrees of freedom and *P* value noted<br>*Give P values as exact values whenever suitable.*
☒	☐	For Bayesian analysis, information on the choice of priors and Markov chain Monte Carlo settings
☒	☐	For hierarchical and complex designs, identification of the appropriate level for tests and full reporting of outcomes
☒	☐	Estimates of effect sizes (e.g. Cohen's *d*, Pearson's *r*), indicating how they were calculated

Our web collection on statistics for biologists contains articles on many of the points above.

Software and code

Policy information about availability of computer code

Data collection	We collected flow cytometry data using FACS DIVA software (version 8.0.1). Images were aquired using ZEN software (version 2.3.) and LAS X software (version 2.0.2). We processed single cell data with CellRanger (10X Genomics) suite (version 3.0.1).

Data analysis	For secondary single cell analsysis we used R software (version 3.5.1) and packages written in R for data analysis and visualization: circlize (version 0.4.10.), clusterProfiler (version 3.10.1), DDRTree (version 0.1.5), hash (version 2.2.6.1), ggplot2 (version 3.3.0), RColorBrewer (version 1.1-2), pheatmap (version 1.0.12), irlba (version 2.3.3), dplyr (version 0.8.5), reshape2 (version 1.4.4), DOSE (version 3.8.2), enrichR (versions 1.0. and 2.1), VGAM (version 1.1-3), tidyr (version 1.0.2), fgsea (version 1.8.0), enrichplot (version 1.2.0), Biobase (version 2.40.0), stringr (version 1.4.0), Rcpp (version 1.0.4.6), scales (version 1.1.0), BiocGenerics (version 0.26.0), ggpubr (version 0.1.9), msigdbr (version 7.1.1), monocle (version 2.6.4), Matrix (version 1.2.-18), magrittr (version 1.5), Seurat (versions 2.3.4 and 3.0.2), viridis (version 0.5.1), patchwork (version 1.0.1),<br>We analysed Epic Array Data with R using RnBeads (version 2.2.). We withdraw REMs from EpiRegio database (version 1) and used Phyton (version 3.8.5) to format data. R version 3.6.1 was used to visualize Epic Array Data with R using packages ComplexHeatmap (version 2.2.0), Gplots (version 3.0.3), RColorBrewer (version 1.1.2) and circlize (version 0.4.9).<br><br>Flow cytometry data was analysed using FlowJo (version 10.7.1)<br><br>Images were analysed using Zeiss ZEN software blue version (version 3.2) and ImageJ (version 1.52g).<br><br>Other data was analysed and visualized using R (version 3.5.1) or Prism (version 8.4.3). Figure panels were created using Inkscape (version 0.92).

For manuscripts utilizing custom algorithms or software that are central to the research but not yet described in published literature, software must be made available to editors and reviewers. We strongly encourage code deposition in a community repository (e.g. GitHub). See the Nature Research guidelines for submitting code & software for further information.

Data

Policy information about availability of data

All manuscripts must include a data availability statement. This statement should provide the following information, where applicable:

- Accession codes, unique identifiers, or web links for publicly available datasets
- A list of figures that have associated raw data
- A description of any restrictions on data availability

R codes for data analysis and visualization are available on GitHub (github.com/TLukas1/NatComm_Tombor_et_al_2020). Raw and processed data for single cell experiments are available at ArrayExpress (E-MTAB9816 and E-MTAB9817). Bulk-RNA sequencing data has been deposited at SRA (PRJNA679225). DNA methylation (RnBeads) output files are available at 10.6084/m9.figshare.13247210. Additional data are available in the source data file or upon request.

Field-specific reporting

Please select the one below that is the best fit for your research. If you are not sure, read the appropriate sections before making your selection.

☒ Life sciences ☐ Behavioural & social sciences ☐ Ecological, evolutionary & environmental sciences

For a reference copy of the document with all sections, see nature.com/documents/nr-reporting-summary-flat.pdf

Life sciences study design

All studies must disclose on these points even when the disclosure is negative.

Sample size	Required sample sizes for in vivo experiments were estimated using power-calculation (p=0.8). For in vitro experiments we estimated sample size based on previous experiments performed by us or others, which we found was sufficient enough to observe significant differences.
Data exclusions	We excluded one animal of Confetti;Cdh5-CreERT cohort from our analysis based on a statistical outlier test (Gibbs test $p < 0.05$).
Replication	Experiments were generally performed in at least 2 independent experiments to ensure reproducibility of the data. Flow cytometry data shown were performed on the same isolated cells used for single cell sequencing and hence not replicated, but confirmed by immunohistochemistry with the same antibodies.
Randomization	Animal have been randomly assigned to treatment cohorts. Cells have been randomly allocated to respective groups.
Blinding	We quantified immunohistochemistry images blinded. Other experiments were non-blinded due to restricted number of researchers and poor feasibility.

Reporting for specific materials, systems and methods

We require information from authors about some types of materials, experimental systems and methods used in many studies. Here, indicate whether each material, system or method listed is relevant to your study. If you are not sure if a list item applies to your research, read the appropriate section before selecting a response.

Materials & experimental systems

n/a	Involved in the study	
☐	☒	Antibodies
☐	☒	Eukaryotic cell lines
☒	☐	Palaeontology and archaeology
☐	☒	Animals and other organisms
☒	☐	Human research participants
☒	☐	Clinical data
☒	☐	Dual use research of concern

Methods

n/a	Involved in the study	
☒	☐	ChIP-seq
☐	☒	Flow cytometry
☒	☐	MRI-based neuroimaging

Antibodies

Antibodies used

We used the following antibodies:
anti-aSMA-Cy3 (C6198, 1A4 monoclonal, Sigma, Lot#GR306964-2)
anti-cleaved Caspase3 (9961, polyclonal, Cell Signaling Technology, Lot#22)
anti-BrdU (560810, monoclonal 3D4, BD Horizon, Lot#9066975)
anti-CDH5 (2500S, D87F2 monoclonal, Cell Signaling Technology, Lot#5)
anti-Fabp4 (15872-1-AP, polyclonal, Proteintech, Lot#00062693)
anti-Fsp1 (07-2274 ,Milipore, Lot#2943792)
anti-GFP (GFP-1010, polyclonal, Aves, Lot#GFP879484)
anti-GFP Fluorescein Alexa 488 (A11090, polyclonal, Invitrogen, Lot#564518)
anti-Mmp9 (ab38898, polyclonal, Abcam, Lot#GR3204084-25)
anti-Pdgfra (AF1062, polyclonal, R&D, Lot#MQO21711)
anti-phoshoH3 (06-570, Milipore, Lot#3319395)
anti-Sm22 (ab10135, polyclonal, Abcam, Lot#GR1926-11)
anti-goat IgG Alexa 647 (A-21447, polyclonal, Thermo Fisher, Lot#1608641)
anti-rabbit IgG Alexa 647 (A-31573, polyclonal, Thermo Fisher, Lot#1964354)
anti-rat IgG Alexa 647 (ab150155, polyclonal, Abcam, Lot#GR3217245-1)
anti-rabbit Alexa 555 (A31572, polyclonal, Thermo Fisher, Lot#2180682)
anti-chicken IgY Alexa 488 (F-1005, polyclonal, Aves, Lot#FGC2917985)

Validation

All antibodies used are commercially available and were validated by the vendors and confirmed by specific labeling of target molecules or cell types:
anti-aSMA-Cy3 (https://www.sigmaaldrich.com/catalog/product/sigma/c6198)
anti-cleaved Caspase 3 (https://www.cellsignal.com/products/primary-antibodies/cleaved-caspase-3-asp175-antibody/9661)
anti-BrdU (https://www.bdbiosciences.com/us/applications/research/clinical-research/oncology-research/proliferation-cell-cycle-and-cell-death/intracellular/cell-signalling-and-transcription-factors/human/v450-mouse-anti-brdu-3d4/p/560810)
anti-CDH5 (https://www.cellsignal.com/products/primary-antibodies/ve-cadherin-d87f2-xp-rabbit-mab/2500)
anti-Fabp4 (https://www.ptglab.com/products/FABP4-Antibody-15872-1-AP.htm)
anti-Fsp1 (https://www.merckmillipore.com/DE/de/product/Anti-FSP1-S100A4-Antibody,MM_NF-07-2274)
anti-GFP (https://www.aveslabs.com/products/green-fluorescent-protein-gfp-antibody)
anti-GFP Fluorescein Alexa 488 (https://www.thermofisher.com/antibody/product/Fluorescein-Oregon-Green-Antibody-Polyclonal/A-11090)
anti-Mmp9 (https://www.abcam.com/mmp9-antibody-ab38898.html)
anti-Pdgfra (https://www.rndsystems.com/products/mouse-pdgf-ralpha-antibody_af1062)
anti-phoshoH3 (https://www.merckmillipore.com/DE/de/product/Anti-phospho-Histone-H3-Ser10-Antibody-Mitosis-Marker,MM_NF-06-570)
anti-Sm22 (https://www.abcam.com/taglntransgelin-antibody-ab10135.html)
anti-goat IgG Alexa 647 (https://www.thermofisher.com/antibody/product/Donkey-anti-Goat-IgG-H-L-Cross-Adsorbed-Secondary-Antibody-Polyclonal/A-21447)
anti-rabbit IgG Alexa 647 (https://www.thermofisher.com/antibody/product/Donkey-anti-Rabbit-IgG-H-L-Highly-Cross-Adsorbed-Secondary-Antibody-Polyclonal/A-31573)
anti-rat IgG Alexa 647 (https://www.abcam.com/donkey-rat-igg-hl-alexa-fluor-647-preadsorbed-ab150155.html)
anti-rabbit Alexa 555 (https://www.thermofisher.com/antibody/product/Donkey-anti-Rabbit-IgG-H-L-Highly-Cross-Adsorbed-Secondary-Antibody-Polyclonal/A-31572)
anti-chicken IgY Alexa 488 (https://www.aveslabs.com/products/fluoresceinated-goat-anti-chicken-igy)

Secondary antibodies have been tested in our experimental conditions to rule out unspecific signal.

Eukaryotic cell lines

Policy information about cell lines

Cell line source(s)

Human umbilical cord endothelial cells (HUVECs) were purchased by Lonza

Authentication	Not authenticated by the authors
Mycoplasma contamination	HUVEC cells were tested and negative for Mycoplasma.
Commonly misidentified lines (See ICLAC register)	There were no misidentified lines used in this study.

Animals and other organisms

Policy information about studies involving animals; ARRIVE guidelines recommended for reporting animal research

Laboratory animals	We used C57BL/6J Cdh5-CreERT2 (Jackson Laboratory, MGI:3848982) or C57BL/6 Col1a2-CreRT2 (Jackson Laboratory, 029235) bred with mTomato/mGFP (mT/mG) (Jackson Laboratory, Stock No. 007676). C57BL/6J Cdh5-CreERT2 were also bred with Rosa26 Confetti mice (Jackson Laboratory, Stock No. 017492). BL6 C57BL/6J wildtype animals (Janiver) were used for immunohistochemisty. Mice were 12 weeks old (AMI time course experiments) when experiment started. Male and females used.
Wild animals	This study did not involve wild animals
Field-collected samples	This study did not involve field-collected samples
Ethics oversight	All animal experiments were executed in agreement with the animal welfare guidelines and German national laws. All animals experiments and study protocols were authorized by the competent authority (Regierungspräsidium Darmstadt, Hessen, Germany).

Note that full information on the approval of the study protocol must also be provided in the manuscript.

Flow Cytometry

Plots

Confirm that:

- [x] The axis labels state the marker and fluorochrome used (e.g. CD4-FITC).
- [x] The axis scales are clearly visible. Include numbers along axes only for bottom left plot of group (a 'group' is an analysis of identical markers).
- [x] All plots are contour plots with outliers or pseudocolor plots.
- [x] A numerical value for number of cells or percentage (with statistics) is provided.

Methodology

Sample preparation	Digestion and isolation of primary cells from murine infarcted hearts. HUVEC cell culture cells trypsinized.
Instrument	BD LSRFortessa X-20
Software	FACS Diva Software and FlowJo
Cell population abundance	For primary cell flow cytometry the analysed population (live cells - singlets - GFP+/FSP1+) was 7.92% or 16.2% of all live cells - singlets - GFP+ cells. GFP+ cells were 10% or 3.27% of the live - singlets population respectively. Live cells were 9.89% or 45.9% of all events measured. We used unstained controls for validation.<br>For HUVEC flow cytometry S-phase cells represented 18.1% or 6.1% of all live cells. Live cells were 91.2 % of all events.
Gating strategy	A detailed gating strategy is included in the respective supplementary figure. Live cells were defined by FSC-A/SSC-A, singlets by FSC-A/FSC-W panels.

- [x] Tick this box to confirm that a figure exemplifying the gating strategy is provided in the Supplementary Information.
